# Equitable and Effective Distribution of the COVID-19 Vaccines – A Scientific and Moral Obligation

**DOI:** 10.34172/ijhpm.2021.49

**Published:** 2021-04-26

**Authors:** Agnes Binagwaho, Kedest Mathewos, Sheila Davis

**Affiliations:** ^1^Vice Chancellor’s Office, University of Global Health Equity, Kigali, Rwanda.; ^2^Partners in Health, Boston, MA, USA.

**Keywords:** COVID-19 Vaccine Distribution, Equal Access, Vulnerable Populations, Unethical Practices, Logistical Capacity

## Abstract

The rapid development of coronavirus disease 2019 (COVID-19) vaccines has not been met with the assurance of an effective and equitable global distribution mechanism. Low-income countries are especially at-risk, with the price of the vaccines and supply shortages limiting their ability to procure and distribute the vaccines. While the COVAX initiative is one of the solutions to these challenges, vaccine nationalism has resulted in the hoarding of vaccines and the signing of parallel bilateral deals, undermining this formerly promising initiative. Moreover, inequity in local distribution also remains a problem, with clear discrimination of minorities and lack of logistical preparation in some countries. As we continue to distribute the COVID-19 vaccines, pharmaceutical companies should share their technology to increase supply and reduce prices, governments should prioritize equitable distribution to the most at-risk in all nations and low-income countries should bolster their logistical capacity in preparation for mass vaccination campaigns.

A year after the detection of the first case of coronavirus disease 2019 (COVID-19), various milestones in the vaccine development process have provided a glimmer of hope. By vaccinating the global population with the approved vaccines, the world hopes to bring an end to the health, economic, social and political crisis caused by the COVID-19 pandemic. The vaccine will not only protect the vaccinated individuals, but it will also help prevent transmission by helping develop herd immunity once 70% of the world population is vaccinated.^1^ However, if efforts are not made to distribute them equitably across the globe, the discovery of the vaccine alone will not guarantee an end to the pandemic. Given the nature of the virus and its global spread, an equitable approach to vaccination is key to developing herd immunity, returning to a state of normalcy with unrestricted travel and cross-border transportation of goods, reversing shrinking economies and most importantly continuing the progress in human development through functioning health and education sectors.

As of April 18, 2021, there are eight COVID-19 vaccines approved for full use in several countries, with five others in early or limited use in some parts of the world.^2^ After performing a close scrutiny of the data from the Pfizer-BioNTech and AstraZeneca vaccine clinical trials to assess its safety, efficacy and quality, the World Health Organization (WHO) has approved it for emergency use.^3^ Countries around the globe that rely on WHO’s approval of drugs and vaccines can now procure and distribute these vaccines to their populations. However, now that the vaccines have been discovered, the dilemma of their manufacturing and equitable distribution is proving to be a more challenging issue than their discovery. The Duke Global Health Innovation Center estimates that the majority in low-income countries will have to wait until 2023 to be vaccinated, twice the time it took to develop, test and approve the vaccines.^4^

This challenge to equitable distribution of potentially life-saving medical and public health interventions is not a novel phenomenon, with the vulnerable in low-income countries often being the last to receive them. Remember that the delayed distribution of available antiretroviral therapy for HIV/AIDS resulted in 330 000 preventable deaths in South Africa between 2000 and 2005.^5^ Other examples include the drugs for multi-drug resistant tuberculosis and vaccines against the novel strain of influenza A (H1N1) in 2009.^6^ Low-income countries are often deemed unfit and unworthy of receiving new drugs and vaccines, which are considered too expensive for them. The core of the issue is the lower value attached to the lives of those in low-income countries compared to those in the global North.

The current distribution of the COVID-19 vaccines is already exhibiting the usual signs of inequity. High-income countries have on average vaccinated 1 in 4 of their populations whereas in low-income countries this ratio is 1 in 500.^7^ As rich nations representing 14% of the world’s population have bought up 53% of the world’s supply of the most promising vaccines including 100% of Moderna’s and 97% of Pfizer’s expected supply for 2021, low- and middle-income countries (LMICs) are turning towards other vaccines.^9^

At the center of all access discussions is cost and affordability. Historically, we have seen the unethical behavior of big pharma in setting unaffordable prices. They sold drugs at multiple times their production cost, as exemplified by the 12 week hepatitis treatment which was sold at $84 000 in the US and more than 15 times their value in some LMICs despite the extremely low production cost of $68-136.^8^ This is not only unethical because it is denying the vulnerable their right to their health but also because a large portion of research and development for these drugs is publicly funded through citizens’ taxes, including the taxes of those who cannot afford the product.^9^ The issue of cost and affordability is also a concern when it comes to the COVID-19 vaccines. Governments of low-income countries are especially at-risk if the price for the vaccines is not subsidized.

To circumvent this challenge, the WHO, the Coalition for Epidemic Preparedness Innovations (CEPI) along with GAVI, the Vaccine Alliance have established the COVAX initiative. The COVAX initiative builds on the principle of equity to provide a safety net to all its 190 member countries by promising to provide coverage for 20% of their populations – with front line workers first to be served – reaching 2 billion doses by 2021.^10^ The promise of COVAX is unlike any other institution we have had in the past as, even before the discovery of the vaccines, it combined the mission of safety and equitable distribution to all regardless of ability to pay. Currently, the COVAX initiative has secured enough doses to protect health and social workers in the first quarter of 2021, with 1.3 billion directed towards the 92 economies eligible for the Advance Market Commitment and with more doses underway for the rest of the year.^11^

While COVAX has met its 2 billion goal for 2020, as of April 9, 2021, it will need an additional $3.1 billion to achieve its 2021 targets.^12^ The US’ recent decision to join COVAX might support the achievement of the 2021 funding targets but various obstacles remain as COVAX looks towards delivering vaccines to LMICs. There is widespread fear that the COVAX initiative will not be sufficient to address the needs of low-income countries. It is estimated that 70 poor countries will only be able to vaccinate 10% of their populations as opposed to COVAX’s commitment to vaccinate 20% of member countries’ populations.^13^ Moreover, the majority of developed countries have broken their commitment by signing other bilateral deals with manufacturing companies, undermining COVAX, depleting the supply of vaccines available, simultaneously driving up prices, and joining the camp of vaccine nationalists.^14^

We have seen evidence that countries are hoarding vaccines, with rich nations such as Switzerland amassing more than seven times the amount needed to inoculate their entire population if all the vaccines are approved.^15^ Such behavior has resulted in the depletion of vaccines available for developing countries and has exposed the limits on the production capacity of manufacturing companies. Despite AstraZeneca’s pledge to provide 64% of its vaccines to the developing world, its production capacity will not reach more than 18% of the world’s population.^16^

To ensure that the vulnerable in need have access to COVID-19 vaccines, it is imperative that manufacturing companies decentralize and share their technology while keeping their intellectual property but producing at lower prices. An example is the agreement between AstraZeneca and the Serum Institute of India to produce the vaccine for developing countries at a lower price.^17^ Therefore, such partnerships not only increase global knowledge and know how, but also increase the supply available, push down the price for the vaccines and ultimately save lives – a goal that should be the motivation of research in the pharmaceutical industry.

However, while equity at the global level is key, equitable distribution within countries is also a critical need.^18^ In the United States the initial wave of vaccination has reinforced the structural discrimination of minorities that led to the disproportional impact of COVID-19 on historically underserved communities. Although the racially diverse frontline workforce was the first priority for vaccination, Black Americans have been significantly underrepresented. Black, Hispanic and Native Americans in the United States are dying of COVID-19 at three times the rate of White Americans and should be the priority for vaccination.

Another significant obstacle to equitable distribution of vaccines within countries is lack of logistical preparation. Some developed countries such as the United States and France have faced this problem. All governments need to take the responsibility to organize internal vaccine distribution with the support of multilateral institutions such as WHO and GAVI because there is a long way to go from the arrival of the vaccines at national airports and the vaccination of each and every individual at risk within the countries. The different vaccine types require varied logistical capacities. Note that the Pfizer vaccine needs to be stored at -70 to -80°C, presenting a significant challenge especially in many low-income countries.^19^ If countries are not prepared to distribute the available vaccines to their citizens, then planning and budgeting for equitable distribution to countries at the global level is of no value. Countries such as Rwanda have made this a priority by preparing the cold-chain capacity and distribution logistics prior to the arrival of the vaccines.^20^ The country had also designed a prioritization and allocation guide according to which it is serving the most at-risk. This level of preparedness in anticipation of the COVID-19 vaccines is key to the ethical and efficient management of their distribution when they are available and should consequently be implemented in all countries across the globe.

 As we continue to search for solutions to this pandemic, maintaining prior commitments to global solidarity and access through initiatives such as COVAX is clearly a prerequisite to stop this pandemic. National governments should be accountable for preparing the needed logistical measures to ensure an equitable, ethical and effective distribution, with priority given to the most at risk. We all need to understand that given the nature of the virus and our global eagerness to return to a state of normalcy, the bottom line is that no one will be safe until we all are.

## Ethical issues

 Not applicable.

## Competing interests

 Authors declare that they have no competing interests.

## Authors’ contributions

 Conception and design (AB, KM); Drafting of the manuscript (AB, KM, SD); Critical revision of the manuscript for important intellectual content (AB, SD).

## Authors’ affiliations

^1^Vice Chancellor’s Office, University of Global Health Equity, Kigali, Rwanda. ^2^Partners in Health, Boston, MA, USA.
